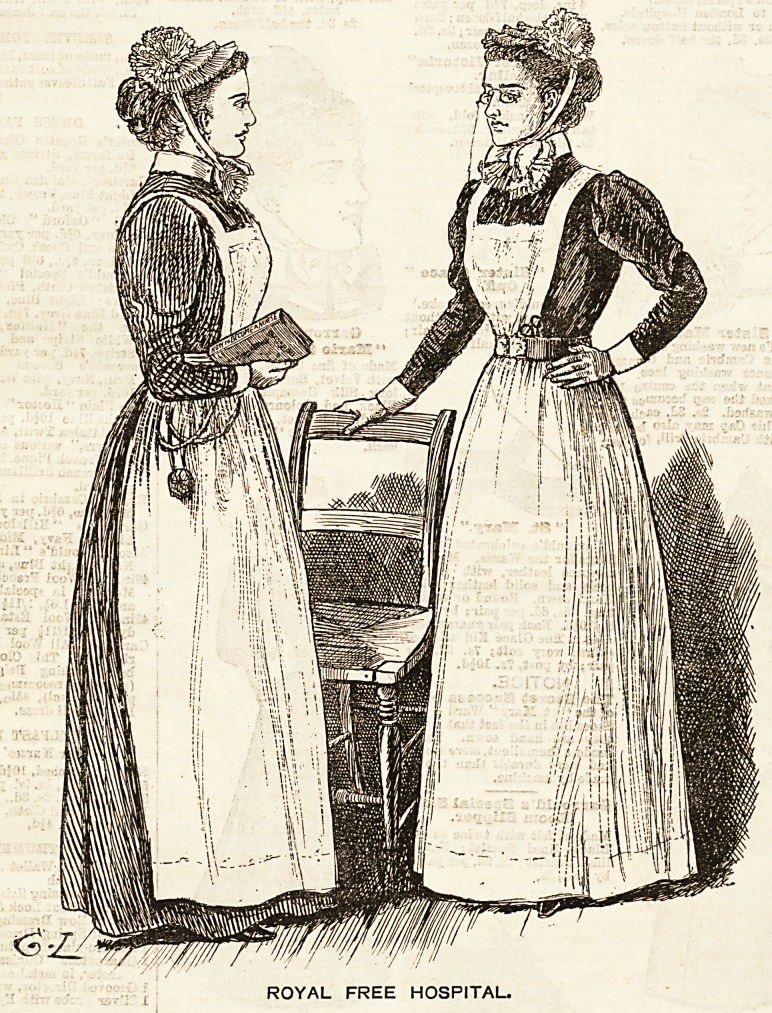# The Hospital Nursing Supplement

**Published:** 1895-07-13

**Authors:** 


"The Hospital" Nursing Mirror.

Being the Extra Nursing Supplement of "The Hospital" Newspaper.

[Contributions for this Supplement should be addressed to the Editor, The Hospital, 428, Strand, London, W.C., and should have the word "Nursing" plainly written in left-hand top corner of the envelope.]

### News from the Nursing World.

### "THE HOSPITAL" CONVALESCENT FUND.

"Nurse is looking and feeling decidedly better since she came here," writes the head of the convalescent home to which an invalid was sent by means of The Hospital Convalescent Fund. Having undergone an operation, rest and change were afterwards considered essential to the nurse's restoration to health. There seemed, however, little chance of her securing them until her case was brought to our notice by a matron, who knew all the circumstances. Another application has just been received from an experienced nurse, devoted to her calling, whom a period of ill-health has incapacitated from work. Her savings are exhausted, and the change of air which may possibly help her to regain her normal strength is, to her great joy, put within her reach by The Hospital Convalescent Fund. All who are enjoying the prospect of pleasant holidays may well spare a thought to those who are poor in pocket, as well as health. Increased subscriptions will add to our power to help many brave workers, whom a little timely rest, free from expense or anxiety, will rescue from a serious "breakdown." We have to acknowledge, with thanks, 5s. as an annual subscription from Miss G. Nelson, and also a donation of 20s. from Miss Helen Rodgers.

### SCHOOL OF MEDICINE FOR WOMEN.

The annual distribution of scholarships, prizes, and certificates at the London School of Medicine for Women took place on the 9th inst. The attendance of visitors was large enough for the garden and verandah to supplement agreeably on so hot a day the accommodation provided by the large class-room. The Dean, Mrs. Garrett-Anderson, M.D., who was in the chair, with the Duchess of Portland and the Countess of Strafford on either side of her, made a short speech, which was followed by the treasurer's report. The Duchess of Portland then distributed the prizes to the students, after which she gave a charming little address, in which she declared herself deeply interested in the work of medical women. She spoke of the value of their services, not only in India, but in England, where she thought that hospitals for women and children should certainly be officered by them. Sir James Stansfield proposed a vote of thanks to the Duchess, which was seconded by Mrs. Keith, M.D. Mrs. Garrett-Anderson thanked Sir James Stansfield in the name of the council for all he had done for the school. She then read and presented to him an address on the occasion of his retirement from public life. In returning thanks to the council, Sir James Stansfield modestly made light of his services to the cause of medical women; but both in the House of Commons and also in association with the Royal Free Hospital his good offices are never likely to be forgotten.

### THE LONDON HOSPITAL.

The annual examinations of the probationers at the London Hospital have again been conducted by Dr. Hayward, of Haydock, who has expressed himself well pleased with the result. There were sixty candidates, forty-nine of whom passed. Of these eighteen were marked "very satisfactory," and the remainder "satisfactory." The first prize was won by Probationer Petty, the second and third by Probationers Lyttel and Ashbe. Honorary certificates were gained by Probationers Holcroft, Mays, and Eva Fry.

### "HER MAJESTY'S HOSPITAL," STEPNEY.

On Monday, July 8th, the Crown Prince of Denmark paid a private visit to Dr. Barnardo's Homes at Stepney. After inspecting the workshops of the different trades, he went round the wards of "Her Majesty's Hospital," and spoke most kindly to the patients, showing great interest in the cases. Before leaving the Crown Prince wrote in the visitors' book, "I have been very pleased to visit the hospital, where I found everything in the best order."

### GOOD BANDAGING APPRECIATED.

It is said that the neatness of the bandage applied to the fractured jaw of a native first drew the attention of the Ameer of Afghanistan to Dr. Lilias Hamilton. He asked who had done it, and the man replied, "The lady who cures every one." The high esteem in which Miss Hamilton is held by the Ameer's household is shown by his refusal to let his young son come to England unless she accompanied him. All the sanitary and hygienic arrangements are reported to be left entirely in the hands of this lady doctor, in whom the native men as well as the women place implicit confidence.

### LEISURE IN THE WORKHOUSE.

Leisure in the workhouse infirmary is mostly a dreary time for old and feeble inmates. Those more happily placed can do much to lighten its burden, and the Countess of Meath has set a happy example by encouraging the use of the needle amongst those incapacitated from work, but wearying for occupation. To ladies who have leisure it is but a small task to devote a few hours to giving the necessary instruction, and they will reap genuine pleasure in seeing how happily the time can be passed by the aged, proud of the powers to accomplish work which would not shame more practised hands. We have just seen a table-cloth, skilfully worked by an old man of seventy-five, an inmate of the Paddington Infirmary, which found a ready purchaser, pleased to possess an article of such interest. Individual workers do not receive payment in money, the sum raised at the annual sale being devoted to the purchase of materials for the ensuing year, and also to tobacco for the men and small gifts for the women. In the winter the entertainments—of teas, &c.—are got up for the patients out of the surplus funds. The work is entirely voluntary and exceedingly popular, the bright colours and varieties of material offering attractions which the patching, mending, and making of ward linen and clothing can hardly compete with. Yet it is necessary that, in the women's wards at any rate, the obligation for inmates to help with the institutional sewing should not be lost sight of.

### LADIES' WORK AT EXETER.

The stalls exhibited in the streets of Exeter, on the occasion of the ladies' collection for the Exter and District Hospital Saturday Fund, were unusually pretty. A lavish display of gay flags and awnings was supplemented by a wealth of flowers, fruit, and growing plants sent by generous donors to be sold. These contributions appear to have resulted in substantial benefit to the fund.

### THIRD AND FOURTH THOUSAND.

A number of nurses have expressed their intention of staying a night in London when they come to town for the reception at Marlborough House, which "Our Princess" has graciously promised to hold for the third and fourth thousand of the Royal National Pension Fund Nurses. Information as to nurses' clubs and other establishments where breakfasts and beds can be secured at moderate rates, should be addressed (by letter only) to the Secretary, 28, Finsbury Pavement. The date and general arrangements for this eagerly anticipated day will be duly announced in our columns.

### INTELLIGENT GUARDIANS.

The Scarborough Guardians have set a good example by the prompt attention they have given to the understaffing of their infirmary as soon as the head nurse called their attention to it. She has been on duty herself for fifteen hours daily, with seventy-three patients, her usual assistant having had to take night duty with bad cases. A proposal to postpone dealing with the matter was withdrawn and the Guardians sanctioned temporary help being immediately secured, and another probationer being added to the staff.

### STILL WITHOUT NIGHT NURSES.

With an average of a hundred and thirty patients i their workhouse infirmary, the Coventry Guardians are content to again defer the provision of a night nurse for the sick and infirm. Months ago the subject was brought to their notice, but they still see no reason to act, because the patients "do not complain." Those members of the Board in favour of the appointment of a trained night nurse are out-voted, although (as reported in the local press) they seem to have proved the urgent need for one. The dying are seldom able to "complain," and the greater proportion of deaths take place during the night. It is not only the lack of nursing that forms the sadness of these death-beds; "bad language and very unseemly scenes had taken place in the wards because there was no one in authority there at night," said a Guardian, speaking "on good authority." Another gentleman is reported to have remarked that "he did not think there was a Guardian present who would care to be left in the charge of paupers." Yet eleven Coventry Guardians still support existing arrangements, preferring to await complaints which may not improbably reach them in due course through a coroner's verdict.

### QUEEN'S NURSES APPRECIATED.

The Drogheda Branch of Queen Victoria's Jubilee Institute has issued its first annual report, which appears to be most satisfactory. Much appreciation has been shown of the services of the two Queen's Nurses already at work amongst the poor. A third nurse would find plenty of work, and it is hoped the subscriptions will make an increase of the staff shortly possible.

### NURSING (?) IN AN IRISH WORKHOUSE.

The revelations recently made regarding the nursing department at Ballycastle Workhouse Infirmary are of a somewhat appalling character, as reported in the Irish press. Neither the matron nor any other woman employed there by the guardians appears to possess any midwifery knowledge. The male lunatics' attendant is an almost blind pauper, who is past sixty years of age. A female pauper administers medicines, although she cannot read; and other of the attendants are reported by the Medical Inspector as so ignorant that he does not think "they would be capable of being taught." No less than three of these unfortunate women are in charge of wards day and night, and are severally reported as the mothers of illegitimate children. The inquiry which has brought to light these facts was made in consequence of statements made by the medical officer, Dr. O'Connor. He called the attention of the Guardians to the condition of the nursing in February last, thus endorsing the opinion given by the Local Government Board inspector. In spite of the representations made to them, the Guardians "expressed themselves satisfied that the present staff was quite sufficient, and that the patients were particularly well attended to." The Local Government Board, however, deemed it advisable to obtain an opinion from their own medical inspector on the subject, and his investigations have shown the low standard of intelligence and morality required of those entrusted with the nursing of the sick and helpless by the Guardians of the poor at Ballycastle Workhouse.

### WOMEN'S WORK IN PARIS.

The obstacles which have hitherto opposed themselves to ladies desirous of obtaining in Paris hospitals a certain amount of training in nursing led to the formation of a scheme by which the required instruction could be secured. The building of a hospital was undertaken by the Association des Dames Françaises, and one pavilion is already completed. The hospital will accommodate twelve medical and twelve surgical patients in times of peace, but some 120 beds will be available should war or an epidemic break out. It is apparently designed by means of the new hospital to give all women opportunities for becoming reliable nurses in times of war, and the scheme has the support of Madame Felix Faure, by whom the foundation-stone was formally laid. Although facilities for the study of medicine are given to the women of France, no opportunities for systematically learning the art of nursing seem to have fallen to their lot.

### SHORT ITEMS.

The House Committee has been asked to enquire into the whole question of the nursing at Gainsborough Workhouse.—The annual report of the Harborne Nursing Society shows that 4,445 visits were paid to 98 patients by the nurse in the course of last year.—The Shipton Board of Guardians have agreed to subscribe five guineas per annum to the Nursing Association for periodical visits to be paid to the workhouse by a nurse.

### Elementary Anatomy and Surgery for Nurses.

By W. McAdam Eccles, M.B., M.S., F.R.C.S., Lecturer to Nurses, West London Hospital, &c.

XXV.—THE NERVOUS SYSTEM (*continued*).

The *spinal cord* itself is a cylinder of nerve matter lodged in the canal formed by the arches of the vertebræ; but in the adult it only reaches as far as the lower border of the first lumbar vertebra, below which the canal only contains nerve trunks derived from the cord, forming a bundle called the cauda equina, or the horse's tail. (See fig. 34.) The weight of the spinal cord is about one ounce, and its length some eighteen inches. It is covered like the brain by three membranes, again called the dura mater, arachnoid, and pia mater. In the cervical and lower dorsal or lumbar regions the cord is much larger than elsewhere, owing to the large size of the nerves coming off from it to pass to the arms and legs. (See fig. 34.) In transverse section of the cord there will be seen an anterior and posterior median fissure, which practically divide it into two halves, joined, however, by a commissure, in the centre of which is a minute canal, the central canal of the spinal cord which contains fluid like the ventricles of the brain. The grey matter of the cord is internal to the white, and disposed somewhat in the form of the letter H, the projecting parts being called cornua, anterior and posterior, the limb uniting them being the commissure. The white matter is grouped in bundles around the grey matter. (See fig. 35.)

Fig. 34.—The Spinal Cord.

From the under surface of the brain twelve pairs of bundles of nerve fibres are given off; these are spoken of as the cranial nerves, and some of them are nerves which pass from the organs of special sense to the brain. The cranial nerves are numbered from before backwards. The first pair are the olfactory nerves, or the nerves of the special sense of smell. The second are the nerves of the sense of sight—the optic. The third are distributed to most of the muscles which move the eyeballs. The fourth go to a special muscle in each orbit. The fifth are very large nerves, and contain both sensory and motor fibres; each divides into three large trunks, which pass to the skin of the face, the teeth, and muscles of mastication; part of the nerve is also possibly concerned in the special sense of taste. The sixth go to another muscle of the eyeball. The seventh are the facial, which supply all the muscles of expression, &c. The eighth, the auditory, are the nerves of hearing. The ninth, called the glosso-pharyngeal, supply the muscles of the pharynx, but the greater part of the nerve is a nerve of taste. The tenth are again large nerves, known as the pneumo-gastric, and have a very wide distribution, sending fibres to the larynx, lungs, heart, stomach, and liver. The eleventh supply certain muscles in the neck. The twelfth, or hypo-glossal, are the motor nerves of the muscles of the tongue. (See fig. 36.)

From each anterior horn of the grey matter of the spinal cord there proceeds a bundle of nerve fibres which is designated the anterior root of the spinal nerve. In like manner to both posterior horns a posterior root passes, and developed upon it is an enlargement containing nerve cells and called a ganglion. These two roots soon join together in the intervertebral foramen to constitute one of the thirty-one spinal nerves derived from each side of the spinal cord. The fibres coming from the anterior root of the cord pass to muscles and are called motor; those entering the posterior root come from the skin, &c., and are called sensory. See fig 35.)

Fig. 35.—Section of Spinal Cord, with Roots of Spinal Nerves—Front View. 1, 1, Sensory Root; 2, 2, Combined Nerve Trunk; 3, 3, Motor Root.
(N.B.—No enlargement or ganglion should appear on the anterior root, only on the posterior.)

The nerves of the *cervical region* of the cord are eight pairs. From the third, fourth, and fifth is derived the important motor nerve of the diaphragm known as the phrenic. The fifth, sixth, seventh, and eighth cervical, with the greater part of the first dorsal nerve form a plexus styled the brachial plexus, the terminations of which pass to the

Fig. 36.—The Cranial Nerves.

upper limb. The chief branches derived from it are: (1) The ulnar, running down the inner side of arm, and supplying muscles in the forearm and hand, as well as the skin of the little and half of the ring fingers. Behind the internal condyle of the humerus the nerve is popularly known as the funny bone. (2) The median in the middle of the arm also supplies muscles in the forearm and hand and the skin of the palmar aspect of the 3½ outer digits. (3) The musculo-spiral, running behind the humerus, supplies the extensor muscles of the upper limb, and by its radial branch the skin of the dorsal surface of the 3½ outer digits.

The *dorsal* nerves are twelve pairs, and are called intercostal. The *lumbar* five pairs, and the *sacral* five pairs, form two plexuses, which send branches to supply chiefly the lower limb. A large nerve on the front of the thigh from the lumbar plexus is called the anterior crural, and a still larger one from the sacral plexus at the back of the thigh is known as the sciatic. Both supply muscles and skin. The last pair are the coccygeal nerves.

### Hints on the Private Nursing of Mental Cases.

By a Nurse.

So many mental cases are nursed at home that a few hints may be acceptable to those about to undertake the care of such patients. The mental nurse should have at least one year's general hospital training, and a thorough knowledge of massage. It is most essential that she should also possess great patience and tact, an even temper, and great firmness of character. With these she need not be afraid of her patients, even on their worst days, and, indeed, she must never show fear.

The first thing a nurse should do on arriving at her destination is to make herself thoroughly acquainted with the house, as patients may wander about; they cause harm to themselves or others if alone in places with which they are unfamiliar. The apartments set aside for patient and nurse should, if possible, consist of one sitting-room (two would be better) and a bed-room for the patient and one for the nurse leading out of it. If this latter arrangement cannot be made the nurse must sleep in the room with her charge. There should be free access to w.c. and bath-room—in the former the plug being so arranged that the patient cannot pull it up, for the habits of these mental cases want very strict watching, and "bad days" are brought about by constipation or anuria. If the habits of the inmates of asylums were more closely watched there would be fewer disturbances and less need for single rooms to be resorted to.

In a private house, if the plug is not already arranged, it would be wise to suggest a chain and padlock to make it secure where there is an upward action, and the chain can be removable where the cistern is above. All taps should be locked in bath-rooms and all windows blocked, allowing, of course, ventilation at top and bottom.

At the first meal a nurse should note how a patient eats. If fast, then food which can be easily digested must be given. Later on, by a little persuasion, the patient will learn to eat more slowly, and then the diet can be varied. All mental cases (who will eat at all) are fond of good things, and may be encouraged by the promise of a favourite dish; but the nurse must never promise what she cannot fulfil. The next duty is the undressing and bathing, in many cases the latter is best done at night, for a warm bath often soothes a patient and produces sleep. A thermometer must always be used, and, if possible, the nurse should remain in the room. She will note bruises, cuts, or deformities, and pay particular attention to the shape of the abdomen, and it is always better to report too much than too little to the doctor. Measure the quantity of urine passed in 24 hours, and, if desirable, keep a specimen. It is an excellent thing for the nurse to understand testing. Temperature should be taken if it does not unduly excite the patient, and the nurse will constantly find it but little over 96 in nervous cases, with a pulse of 78. Massage may be ordered to improve the circulation in these cases.

The nurse should pay great attention to all her patient says and does, find out delusions but never argue concerning them. Never forget that delusions are realities to the insane, therefore to laugh at them is unkind.

It is well to find out any favourite occupation, games or taste for music. Singing is a favourite recreation, and a nurse may be called upon to play accompaniments.

A nurse must find out all she can about the disease, if hereditary, &c., also the character of her patient before illness.

Puerperal insanity and epileptic mania must be tended by experienced nurses, but melancholia, mental stupor, dementia in the form of senile decay, and dipsomania often fall to the care of less experienced people.

Melancholia is better nursed away from the patients' friends, for if this were done from the commencement of an attack more cures might be effected, or at least the attack made shorter.

This may sound harsh, but anyone who has seen a patient of this class dragged from a chair to the sofa, from room to room, told to hold her head up, to keep her hands still, &c.; made to answer hundreds of questions; to eat more than she wishes, and so on, will agree with me. Yet, needless to state, all this is done from the kindest motives, mistaken kindness in such cases, for there is much nerve exhaustion, and comparative isolation and rest will do much for the patient. Constipation causes much of the mischief, and must therefore be attended to.

Cascara sagrada is a good aperient, and when the doctor orders it, it is always better to administer the medicine openly. It should not be given in food. Be sure that the medicine is really swallowed, a very common trick being to retain the dose in the mouth, and, when nurse's back is turned, to empty it on the handkerchief, dress, &c.

In cases of melancholia there is often a tendency to suicide. Life becomes a burden, and the thought of ending it is ever present with them. Needles, scissors, matches, pins, &c., should be kept locked up. The nurse can leave her box open whilst she is at work, but should lock it or take it with her if she leave the room. The patient must not fancy that it is locked on her account, but only for tidiness, and it is well to accustom the patient to return a needle, thimble, or scissors she may have been using. Only one needle should be given out at a time, and in marked suicidal cases, of course, even this is not allowed.

Encourage the patient to take an interest in various things, but never force her, and do not excite her if it can be possibly avoided, but strive to secure plenty of rest for her.

With regard to massage given after a warm bath at night, it is better to do the head first, as it frequently soothes the patient, and she will let the nurse massage the rest of the body after. When the rubbing is first commenced, it is better to leave the feet until last, as a tickling sensation is apt to excite unduly, and then no more rubbing can be done.

In melancholia it is, perhaps, better that the nurse should sleep in the patient's room, and it is a good plan to have a night light, using a red glass shade, as it is soothing to the patient on account of the warm look it gives to the room.

All mental cases feel the cold, so the room should be kept warm, and warm clothing used.

Doors should be locked as little as possible, but at night a nurse should lock the bed-room door, placing the key where the patient cannot get it.

Never show distrust towards your patient, yet always keep vigilant watch.

Melancholia is frequently the forerunner of insanity, but is in some cases curable. The nurse has much responsibility, and must cheerfully work on, and not allow herself to get disheartened if the patient has a relapse.

### Everybody's Opinion.

[Correspondence on all subjects is invited, but we cannot in any way be responsible for the opinions expressed by our correspondents. No communications can be entertained if the name and address of the correspondent is not given, or unless one side of the paper only be written on.]

### RECEPTION AT MARLBOROUGH HOUSE.

Mrs. Nichol, secretary of the Trained Nurses' Club, 12, Buckingham Street, Strand, writes: We shall be so glad to have it made known through The Hospital that on the day when her Royal Highness the Princess of Wales receives the third and fourth thousand Pension Fund nurses our five club-rooms will be placed at the service of any nurses liking to use them. It will probably be convenient to many from the country to know of so central a place where they can come to rest, or to put on their caps and aprons. We shall also be pleased to furnish addresses where bed and breakfast can be secured at a moderate charge.

### QUEEN'S NURSES.

Miss Berwick, Queen's Nurse, Assistant Superintendent, writes from Glasgow: I beg to draw your attention to an error in your notice of names entered on the roll of Queen's Nurses. Six nurses serving in Glasgow are entered under Edinburgh. The Glasgow list should be as follows: Margaret A. McBean, Henrietta Gordon, Jessie A. Wood, Mary B. Tennant, Jemima H. MacNaughton, Jessie Currie, Annie D. Hamilton, Margaret D. Hempseed, and Sarah Wright.

[The list of nurses inserted last week was officially communicated to us.—Ed. T. H.]

### METROPOLITAN NURSING ASSOCIATION.

Miss Amy Hughes, late superintendent of the Central Training Home, Bloomsbury, writes: May I draw attention to a statement which might prove misleading with regard to the munificent bequest left by the late Mr. Durham, F.R.C.S., to form a perpetual fund for providing trained nurses for the sick poor of Greenwich? In an account given by the *Daily News*, June 29th, it is stated that "the trustees shall be at liberty to adopt the plan of nursing followed by the London Bible and Domestic Female Mission, of 2, Adelphi Terrace, Strand, whose nurses are at work in all the districts of the Metropolitan and National Nursing Association, of 23, Bloomsbury Square." This seems to imply that these nurses are working in concert with or under the auspices of the M.N.N.A. This is not so in any way, the two associations being absolutely distinct, and working on very different lines. That the two sets of nurses work in the same districts is a question of local geography, as the area nursed from the Central Training Home for Queen's Nurses, 23, Bloomsbury Square, is a very large and densely-populated one, and several local missions which employ nurses are engaged in it. The work of the Queen's nurses differs from that of any of these in being absolutely unsectarian, is mainly under the direction of the resident medical officers of the numerous endowed and provident dispensaries which exist in that area, and also includes all the externe maternity cases for St. Bartholomew's Hospital by special arrangement. All the Queen's nurses are fully trained in hospital work, and work under the supervision and inspection of a trained superintendent and the inspector of nurses to the Queen's Institute. This point of inspection is a special feature of the work of the Queen's Institute, as it keeps the nursing at a uniform standard, and prevents the almost inevitable deterioration which comes when nurses, however good, are left without trained supervision.

### Wants and Workers.

Can anyone tell me of a farmhouse, convalescent, or holiday home for ladies (nurses or otherwise) on the West Coast, near Blackpool or St. Anne's?—*Bess.*

### Care of the Sick in Alexandria and Cairo.

### III.—THE GREEK HOSPITAL.

Although built specially for the Greek community this hospital does not refuse help to any who stand in need of it, whatever their nationality or disease. It is an elegant building, with wide, cool corridors and pretty terraces, and it stands in the midst of a garden. The doctor in charge thinks highly of the system of hospital nursing in England, and it was by his desire that three English sisters undertook the nursing. Those in charge at the time of our visit had all been trained at Westminster. Their uniform consists of rough, stiff brown holland dresses, with white caps and aprons. They wear something softer when present at operations, of which there are many, owing to the number of accident cases admitted; but fever and ophthalmia chiefly fill the beds, and in summer the accommodation for a hundred patients is all in use. Each patient pays three francs a day for a bed in a ward, five for one in a small room, and ten for a good-sized single room. The men's corridor looked extremely pretty, with polished marble floor, and ferns and plants in pots scattered about. The women's was bare, but the sister said the plants were only temporarily removed thence. The mattresses had very good wire springs. In one ward lay the Italian wife of a Greek, and in a cot at the foot of her bed was her child, evidently a great pet in the hospital. In a small room we saw a woman, said to be incurable, who persisted in doing her own washing, which she festooned round the window; this was rather a trial to the nurses. There are separate sisters and wards for infectious cases. The well-arranged operating room has a room attached where the more complicated dressings are carried out. The sisters consider the Arabs clever at nursing, and say they do not as a rule find them unreliable. The sisters go off duty every day for three hours—the one who takes the night duty finds it somewhat lonely. The sitting-room for their use is pleasant.

### Novelties for Nurses.

### NEW "HOLDFAST" SYRINGE.

Nurses will have cause to be grateful to Messrs. Bailey for the latest admirable invention of an enema syringe with rubber suction end. It is only necessary to press the lozenge-shaped end against the basin to cause it to adhere tenaciously. Thus the nurses' attention need no longerbe directed to the position of the syringe, which can be used freely and conveniently, and the whole of the solution utilized without trouble. The absence of all metal renders the syringe a safe medium for perchloride of mercury injections. The prices are very moderate. The syringes can now be obtained from 38, Oxford Street.

### Presentation.

On her departure from the Great Yarmouth General Hospital Miss Bowman, who had held the post of matron there for nearly four years, was presented by the committee with an illuminated address. The honorary staff gave her a silver salver, and from the nurses she received a china tea service and illuminated address. Miss Bowman's departure is much regretted by many friends, whose good wishes follow her to her new work at Macclesfield Infirmary.

### Minor Appointments.

Uxbridge Joint Hospital for Infectious Diseases.—Miss E. I. de Traine has been made nurse matron of this hospital. She received her training at Salisbury Infirmary, and we wish her success in her new work.

### Holidays and Health.

[Readers of The Hospital in need of information about health resorts at home or abroad, or desirous of aid in forming holiday plans, are invited to send queries to Editor, 428, Strand, W.C. (marked "Travel" on outside of envelope), which will be answered under this section.]

### ITALY IN SUMMER.

You need not be deprived of a visit to Italy because August is your only month. There are many places where you may enjoy freedom from excessive heat, or even, if that is desired, the luxury of an artificial winter. Large towns must certainly be avoided even for one night's stay, as travellers from purer air are specially liable to the malaria, and do, indeed, frequently imbibe the poison *en route* and develop fever many days later in localities above suspicion. Yet it is very easy in Italy to fall into the error of overdoing the dread of heat. In places like Vallombrosa and Abetone, several thousand feet above the sea level, the climate at its best is too cool to count as real summer, and at its worst, with cold mist and half-melted snow runs a Scotch November hard for sheer discomfort. Lower down in the Pistoiese Apennines a bewildering choice of charming sojourns lies at the disposition of the summer visitor. Two hours' journey from Florence, passing Pistoia, and beyond that up through some forty tunnels, Pracchia is reached, the highest point to which the railway has been carried, and itself a charming little place. Many of the villages in these mountains are perched on the crest of some apparently inaccessible crag, having grown up round the robber fortresses, which gave the whole region an ill-sounding name in mediæval history, but have now long since been transformed into peaceful summer villas. They command magnificent views, extending on the higher points far away to Pistoia, outstretched on the gleaming plain which looks towards Florence. The slopes are covered with chestnut woods, which furnish the principal food of the peasants, and the sight of all these trees in flower in early summer is one not easily to be forgotten. The peasantry, half-starved and over-worked as they are, have many gentle qualities, and will well repay an effort to make their acquaintance, if only by the sound of the singularly pure Italian which is their common language. The roads, engineered under the direction of Napoleon, are some of the finest in the world, and their masterly construction by the aid of continual viaducts and bridges, strikes the traveller with endless admiration, and adds enormously to the comfort of walking and driving. Up and down among the mountains, often remote from other habitations, pensions abound. The purely Italian ones will be found considerably cheaper, and the food more distinctive, although certain elements of comfort must not be expected. A residence like this among Italians in the heart of the country will convey to you more of the true spirit of Italian life, the spirit of Virgil's eclogues, of Petrarch and Ariosto, than months of sight-seeing in Florence or Rome. San Marcello, reached by carriage from Pracchia (or from Pistoia if you dislike tunnels), forms good headquarters; in a primitive way it is getting to have something of the aspect of a fashionable resort, and Cutigliano, high up among the chestnut woods, offers more perfect seclusion. The baths of Lucca lie within easy distance. The air is delicious, but it is well to be provided with a change of warm clothing, for the contrasts of temperature are great, and heavy dews make the evenings chilly.

For those who can brave almost complete solitude, the Italian lakes in July and August are by no means to be despised, and at considerably lower terms than in the season twice the amount of attention in hotels and pensions will be received. Make Cadenabbia your headquarters; except perhaps for three siesta hours in the middle of the day you will find plenty of shade and very agreeable walking. It is a good plan to hire a boat by the week; numberless places of interest are within reach of an easy row, and you may be on the lake till far into the evening, lulled by the distant tinkling of the bells on the fisher-nets, or by some harmonious chorus from the opposite shore of Bellaggio. The oleanders, oranges, lemons, myrtles, and almost every flower that blooms, will be in full beauty, and though the vivid contrasts at midday against the brilliant blue of the lake may strike the tired eye with a sense of exaggeration, you will gain lessons for a lifetime in the meaning of colour.

### Royal British Nurses' Association.

The secretary of the R.B.N.A. asks us to announce: "Notice is hereby given that the annual meeting of members of the Royal British Nurses' Association will be held at the Queen's Hall, Langham Place, London, W., on Wednesday, July 24th, 1895, at twelve o'clock (noon). Agenda—(1) To read and confirm minutes of previous annual meeting. (2) To appoint scrutineers of ballot-papers for election of General Council, 1895-6; (3) to receive annual, financial, and other reports; (4) to elect the General Council for the ensuing year."

### Notes and Queries.

The contents of the Editor's Letter-box have now reached such unwieldy proportions that it has become necessary to establish a hard and fast rule regarding Answers to Correspondents. In future, all questions requiring replies will continue to be answered in this column without any fee. If an answer is required by letter, a fee of half-a-crown must be enclosed with the note containing the enquiry. We are always pleased to help our numerous correspondents to the fullest extent, and we can trust them to sympathise in the overwhelming amount of writing which makes the new rules a necessity. Every communication must be accompanied by the writer's name and address, otherwise it will receive no attention.

### Queries.

(196) *Infectious.*—Are nurses supposed to nurse small-pox in fever hospitals?—*A Companion.*

(197) *Training.*—Do you know of any hospital where I could get two years' general training, and receive uniform and salary during that period?—*Probationer.*

(198) *Account.*—Is it correct for a nurse who has been ill to ask her doctor for his account?—*Anxious.*

(199) *General Training.*—I should be glad to know of a general hospital in which I could get six months' training in return for my services, or one year at a small salary?—*H. G.*

(200) *Monthly Nursing.*—Will you inform me what is customary in the following case: A nurse was engaged at two guineas a week for a confinement in September, an accident in May causing the engagement to be cancelled. What fee can the nurse ask?—*T. F. S.*

(201) *Correspondence.*—Can you put me in communication with correspondents who ask questions in this column?—*S. M.*

(202) *India.*—Where can I learn particulars as to nursing in India?—*Y. Z.*

(203) *Paying Probationers.*—Can you tell me of any chest hospitals where probationers (paying or non-paying) do not begin work before eight a.m.?—*M. H.*

(204) *Syringes.*—Kindly inform me where I can obtain stands for syringes.—*Nurse.*

### Answers.

(196) *Infectious* (*A Companion*).—In those hospitals where small-pox cases are admitted they certainly require nurses. Perhaps your question is intended to find out whether this fever is admitted to all fever hospitals. In that case we answer "No, it is not."

(197) *Training* (*Probationer*).—You should apply to a workhouse infirmary, or call on the secretary of the Workhouse Infirmary Nursing Association, 6, Adam Street, Strand. You would have to wait a long time for admission to any London hospital, and the training is generally three years.

(198) *Account* (*Anxious*).—We do not quite understand your question. If you have been a private patient, of course you should ask the doctor what you owe him. The generosity exhibited by most medical men towards sick nurses does not obviate the duty and courtesy of making an inquiry.

(199) *General Training* (*H. G.*).—We do not know of any general hospital which would consider services an equivalent for the advantages of six months' training. Read "How to Become a Nurse," by Honnor Morten.

(200) *Monthly Nursing* (*J. F. S.*).—She can ask half the fee.

(201) *Correspondence* (*S. M.*).—We can forward a letter if you like to address it to our care. We do not give up our correspondents' addresses.

(202) *India* (*Y. Z.*).—See "Burdett's Hospital Annual for 1894."

(203) *Paying Probationers* (*M. H.*).—You had better address your query to the Matrons of City of London Hospital for Diseases of the Chest, Victoria Park, E.; Royal Hospital for Diseases of the Chest, City Road, E.C.; or Hospital for Consumption, Fulham Road, S.W.

(204) *Syringes* (*Nurse*).—At Reynolds and Bransome's, 13, Briggate, Leeds.

### Dress and Uniforms.

By a Matron and Superintendent of Nurses.

### THE ROYAL FREE HOSPITAL.

The subject of our sketch this week is a sister and a probationary nurse attached to the Royal Free Hospital. This hospital, as most of our readers are aware, is situated in that busy thoroughfare the Gray's Inn Road. The sister's attire consists in a navy blue merino dress of somewhat dark shade, made with a plain full skirt hemmed round the bottom and gathered into a band at the waist. The bodice is tight-fitting and buttoned down the front, and is attached to the waistband of the skirt. The coat-shaped sleeves fit closely to the arms, but sufficient fulness is allowed to admit of free movement. A white linen apron of ample dimensions is worn over the dress, the bib of which is square and kept in position by straps about two inches wide, which cross behind and fasten at the waist. The cap is made of plain mull muslin, and, when flat, is of a triangular shape. It is trimmed all round with a double row of goffered Coventry frilling, and is drawn into position by a runner at the back, which is adapted to the size of the wearer's head. Strings come from the back edged with frilling, and tie in a neat little bow under the chin. Plain linen turn-down cuffs and collars give the necessary finish to the costume at the neck and wrists. The nurse wears a narrow blue and white striped galatea, the bodice and skirt of which are quite plain, the latter just clearing the ground all round, and turned up with a substantial hem. The apron, cap, cuffs, and collars are similar in shape to those worn by the sisters. The uniform, both indoor and out, is supplied by Messrs. Wallis and Co., of Holborn Viaduct, who are justly noted for the excellence of all the material they provide.

ROYAL FREE HOSPITAL.

## Figures and Tables

**Fig. 34 f1:**
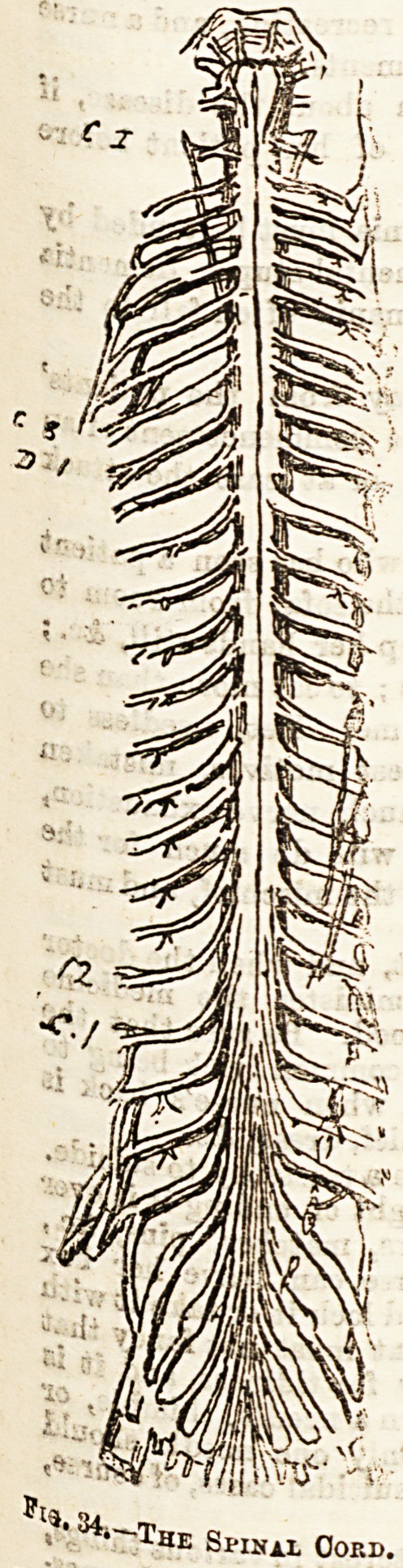


**Fig. 35 f2:**
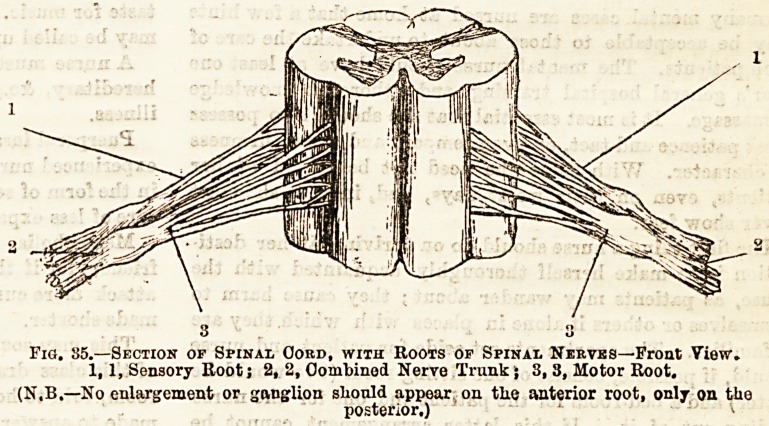


**Fig. 36 f3:**
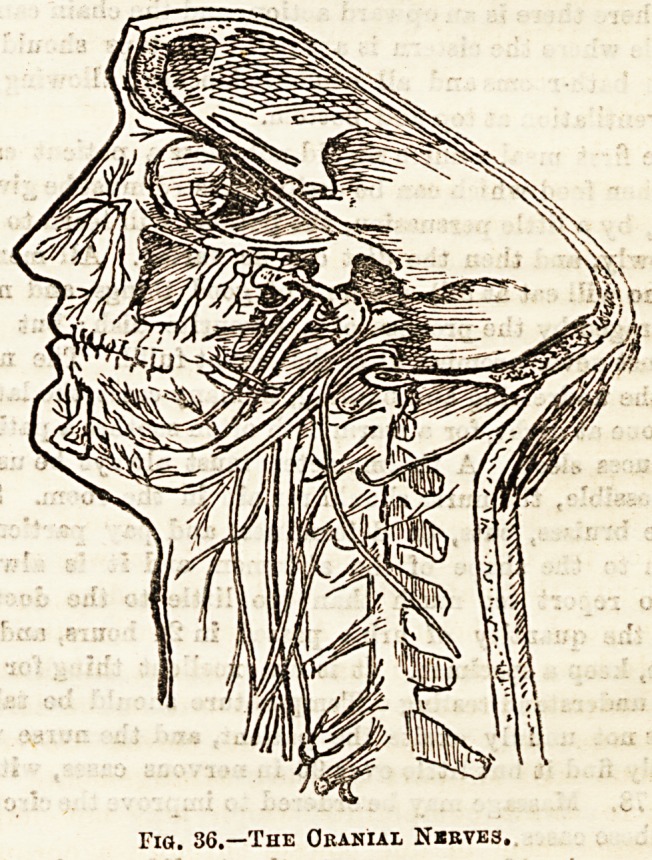


**Figure f4:**